# Avocado consumption and markers of inflammation: results from the Multi-Ethnic Study of Atherosclerosis (MESA)

**DOI:** 10.1007/s00394-023-03134-8

**Published:** 2023-03-22

**Authors:** Feon W. Cheng, Nikki A. Ford, Alexis C. Wood, Russell Tracy

**Affiliations:** 1 Avocado Nutrition Center, Mission Viejo, CA USA; 2 USDA/ARS Children’s Nutrition Research Center, Baylor College of Medicine, Houston, TX USA; 3 Laboratory for Clinical Biochemistry Research, University of Vermont, Burlington, VT USA

**Keywords:** Immune health, Inflammation markers, Avocado, Brain health

## Abstract

**Purpose:**

Since avocado consumption has been linked to a possible reduction in inflammation, we investigated associations between avocado consumption and markers of inflammation in a population-based multi-ethnic cohort [Multi-Ethnic Study of Atherosclerosis (MESA)].

**Methods:**

We used a food frequency questionnaire (FFQ) at MESA exam 1 to capture avocado/guacamole consumption. To calculate daily servings of avocado/guacamole, we used both frequency and serving size data from the FFQ. We classified participants into three consumer groups: rare or never (daily serving ≤ 0.03), medium (0.03 < daily serving < 0.1), and heavy (0.1 ≤ daily serving). Inflammation was estimated by natural log-transformed inflammatory biomarkers (CRP, IL-2, IL-6, homocysteine, fibrinogen, TNF-a soluble receptors). We used multivariate general linear regression models to assess associations accounting for age, sex, race/ethnicity, educational level, income, energy intake, smoking status, physical activity, diet quality, body mass index, and diabetes type.

**Results:**

Among 5794 MESA participants, the average age and BMI were 62.25 y ± 10.26 and 28.28 ± 5.41 kg/m^2^, respectively, and 48% of the sample were men. Participants self-reported as Hispanic (22.30%), Caucasian (39.92%), African-American (25.39%), and Chinese (12.39%). Over 60% had higher than a high school education and 40% made $50,000 or more a year. Regarding avocado/guacamole consumption, 79% were categorized as rare or never, 12% as medium, and 9% as heavy. When adjusted for relevant confounders, there were no significant differences among the three consumer groups for any inflammatory marker.

**Conclusion:**

In this cross-sectional study, we did not find that consumption of avocado/guacamole was associated with levels of inflammatory markers.

## Introduction

Normal immune function is vital to the health of an individual. While various factors affect immune health, nutrition plays an important role [1]. Adequate nutrition is needed for cells to function well, including cells in the immune system. For example, it is well established that malnutrition contributes to chronic inflammation and increases infection risk [1]. While inflammation is part of a healthy immune response and proper wound healing, chronic inflammation (often characterized by mediators such as elevated C-reactive protein (CRP), interleukin 2 (IL-2), interleukin 6 (IL-6), homocysteine, fibrinogen, and tumor necrosis factor-A soluble receptors) is a sign of dysregulated immune health [2–5].

Carotenoids, fiber, several vitamins (i.e., A, D, C, E, B6, B12, B9), and minerals (i.e., zinc, iron, copper, selenium, magnesium) are essential to immune health [1, 6–9]. They have specific roles in innate and adaptive immune responses, which help resolve infection and inflammation. Multiple nutrients often work together to provide proper function at each stage [6]. For instance, several vitamins (A, D, C) and minerals (zinc, iron, copper, selenium) support the innate immune biochemical response, which mediates the acute inflammatory response [6]. Therefore, it is no surprise that a diet high in fruit and vegetables rich in those nutrients, has been shown to decrease inflammation [10].

Avocados contain several vitamins, minerals, and phytochemicals known to support immune health and decrease inflammation [1, 6, 11]. They also contain 8 g of total fat per serving (1/3 medium avocado, ~ 50 g) [11], and high-fat diets/foods have been associated with increased inflammation and poor health conditions [12–15]. However, not all fatty acids have the same effects on inflammation [16–18]. For example, Masson et al. observed that a meal rich in saturated fatty acids increased inflammatory markers. In contrast, a meal rich in unsaturated fatty acids (e.g., polyunsaturated fats) decreased those markers [19]. This is consistent with the results reported by Jiménez-Gómez et al. [20]. When given at breakfast in a 4-week randomized crossover study, they found that olive oil, with a similar fatty acid profile as avocado, was not associated with inflammation, but butter, high in saturated fats, was [20].

Few studies have examined the association between dietary avocado and inflammation. Similar to olive oil, avocados did not lead to higher inflammatory markers in four different studies [21–24], ranging from an acute study [21] to a six-month randomized controlled trial [24]. Acute or longer-term avocado consumption may decrease (or there was a trend for lower) markers of inflammation [25–27]. Uncertainty among these studies may be due to small sample sizes, limited study length, and the fact that most of those studies were not powered to examine markers of inflammation (i.e., secondary outcome). Thus, it is essential to replicate this in a larger, more diverse cohort in a free-living (versus controlled) setting.

We examined the association between avocado consumption and markers of inflammation (CRP, IL-2, IL-6, homocysteine, fibrinogen, and tumor necrosis factor-A soluble receptors) using data from the Multi-Ethnic Study of Atherosclerosis (MESA) cohort. MESA provides a unique opportunity to explore this research question because it is a large, multi-ethnic cohort from six cities in the US, with both men and women, and a wide age range.

## Materials and methods

### Study population

Participants were drawn from MESA. The goals of this cohort were to examine the incidence, associations, and progression from subclinical cardiovascular disease to cardiovascular disease. At baseline, 6814 women and men were free of clinical cardiovascular disease, between 45 and 84 years old, across six study sites in the United States. Approximately 38% of the participants self-reported their race/ethnicity as White, with a representation of African Americans (28%), Hispanics (23%), and Chinese–American (11%) in the original MESA cohort [28].

The baseline examination (Exam 1), which started in July 2000 and was conducted over two years, collected demographic, socioeconomic status, lifestyle, psychosocial, and diet information [28]. Participants also provided blood samples that were analyzed for various biomarkers in different domains (e.g., genetics, inflammation, lipids, hematology, renal function, metabolism, etc.) [28]. All MESA participants provided informed consent, and institutional review boards at all six study sites reviewed and approved MESA protocols [28].

We restricted our analyses to data from MESA Exam 1 since this included both our exposures and outcomes of interest.

### Dietary assessment

A food frequency questionnaire (FFQ) assessed avocado/guacamole consumption. A more detailed description of the MESA FFQ has been published elsewhere [29]. In brief, participants were asked on average last year how often (e.g., rare or never, 1 per month, 2–3 per month, 1 per week, 2 per week, 3–4 per week, 5–6 per week, 1 per day, and 2 or more per day) they had consumed a list of food items (e.g., avocado/guacamole) and the serving size (e.g., small, medium, and large).

To calculate daily servings of avocado/guacamole, we used both frequency and serving size data from the FFQ (e.g., small = 0.5 × medium; medium = 1, and large = 1.5 × medium) [29]. For instance, if a participant consumed 1 small-sized avocado/guacamole (0.5), 5–6 days per week (5.5/7 = 0.79), then the daily serving is 0.5*0.79 = 0.395.

Participants were categorized into three consumer groups: rare or never (daily serving ≤ 0.03), medium (0.03 < daily serving < 0.1), and heavy (0.1 ≤ daily serving), consistent with the avocado consumer tracking study [30].

### Markers of inflammation

Blood samples were processed and stored at − 80 °C utilizing a standardized protocol similar to the Cardiovascular Health Study [31]. Participants were instructed to refrain from vigorous exercise for 12 h, fast for 12 h, and avoid smoking in the morning before the exam. CRP and fibrinogen levels were measured using the BNII nephelometer (N high sensitivity C-reactive protein and N antiserum to human fibrinogen; Dade Behring, Inc., Deerfield, Illinois). IL-2 and IL-6 were measured by an ultrasensitive enzyme-linked immunosorbent assay (Quantikine HS human interleukin-2 and interleukin-6 immunoassay; R&D Systems, Minneapolis, Minnesota). Homocysteine levels were measured using a fluorescence polarization immunoassay with the IMx analyzer (Abbott Diagnostics, Abbott Park, Illinois). Tumor necrosis factor-A soluble receptor was measured using an ultra-sensitive sandwich enzyme-linked immunosorbent assay (Quantikine Human soluble tumor necrosis factor R1 Immunoassay; R&D Systems, Minneapolis, Minnesota).

### Covariates

The following covariates were collected via either interviewer-administered or self-administered questionnaires: age, sex, race/ethnicity, income, energy intake, smoking status, alcohol consumption, physical activity, diet quality [Alternate Healthy Eating Index (AHEI)], Diabetes Type, and medication(s) (hormone replacement therapy, oral steroids, oral anti-inflammatory asthma drugs leukotriene receptor antagonists and inhibitors of lipo-oxygenase, inhaled steroids for asthma, anti-psychotic medications, aspirin) that may influence inflammatory markers. Weight, height, and waist circumference (at the umbilicus) were measured during the clinical examination. Body mass index (BMI; kg/m^2^) was calculated from measured weight and height.

Those (n = 238) with extreme total energy intake (e.g., women: < 500 or > 5000 kcal/day for women and < 500 or > 8000 kcal/day for men) were recoded as having a missing energy intake variable [32]. Calculation of the AHEI in this study was based on a modified version of the AHEI adapted for the FFQ in MESA [33], which has been previously described [34].

### Statistical analysis

We assessed the distribution of the inflammatory markers using univariate descriptive statistics. Because they were not normally distributed (e.g., kurtosis and skewness outside the range of − 1 and 1), we natural log-transformed them to improve the normality of the distributions.

We performed descriptive statistics to show the baseline characteristics among the three consumer groups. We used multivariate general linear models to examine the association between avocado/guacamole consumer groups and markers of inflammation. Model 1 was adjusted for age (45–54, 55–64, 65–74, 75–84), sex (men or women), race/ethnicity (White, Chinese-American, African-American, Hispanic), field center (Columbia University, Johns Hopkins University, Northwestern University, University of California, Los Angeles, University of Minnesota, and Wake Forest University), educational level (less than high school, high school, < 4 years of college, ≥ 4 years of college), income (< $25,000, $25,000– < $50,000, ≥ $50,000), and energy intake (kcals/day, continuous). Model 2 further adjusted for smoking status (never, former, current), alcohol consumption (g/day, continuous), physical activity (metabolic equivalent of task min/week, continuous), diet quality (continuous), BMI (BMI; underweight/normal, overweight, obese I, obese II), Diabetes Type (normal, impaired fasting glucose, untreated diabetes, treated diabetes), and medication(s) that may influence inflammatory markers (yes or no). Model 3 adjusted for covariates selected from the stepwise selection procedure (significance level of 0.05 was used as a criterion for variables to enter or stay in the model)—see Table 2 for the covariates selected for each inflammatory marker. Since waist circumference may be an important covariate, an additional analysis was conducted to control for waist circumference instead of BMI in the final model. Covariates were selected based on previous research showing potential association between these covariates and exposure and/or outcomes [29, 35, 36].

Multicollinearity was assessed but not observed (i.e., tolerance > 0.2 and variance inflation factor ≤ 2.5). Model assumptions were examined using residual analyses (e.g., quantile–quantile plots and residual histograms). We used ANOVA and the Tukey–Kramer procedure to adjust for multiple comparisons. All analyses were conducted with SAS 9.4 (SAS Institute Inc., Cary, NC), and the level of significance was considered at p < 0.05.

### Protocol submission and checklist

Before accessing and analyzing the data, we developed and submitted our study protocol to the MESA Publications and Presentations and Steering Committees. Additionally, we followed the Strengthening the Reporting of Observational Studies in Epidemiology (STROBE) checklist to ensure the quality of reporting.

## Results

Of the 6043 participants who consented to their data for private organizations' use, 249 were excluded from this study because they were missing the daily servings of avocado/guacamole variable. This resulted in 5794 participants for the final analysis. Compared to participants who were included in this study, those who were excluded tended to be slightly younger (mean 60.45 ± 10.39 vs. 62.25 ± 10.26 years), more likely to be African-American (49.80 vs 25.39%), and less likely to be Hispanic (21.29 vs. 22.30%), White (28.92 vs. 39.92%), or Chinese-American (0 vs. 12.39%). They also had a lower percentage of individuals with ≥ 4 years of college (24.47 vs. 35.37%) and ≥ $50,000 income (30.63 vs. 40.16%), a higher percentage of current smokers (25.74 vs. 12.49%), and greater BMI (30.39 ± 6.36 vs. 28.28 ± 5.41 kg/m^2^). However, female sex, physical activity, and diabetes type were similar between those who were included and those who were excluded from this analysis.

As shown in Table 1, 79% of the participants were classified as rare or never, 12% as medium, and 9% as heavy consumers of avocados. Overall, the mean age was 62.25 ± 10.26 years, with about an equal proportion of men (48%) and women (52%). Over 35% had 4 years of college or higher, and about 40% had income equal to or greater than $50,000. The average BMI was 28.28 ± 5.41 kg/m^2^, and the mean AHEI was 42.22 ± 11.66. Most of the subjects were White (39.92%), followed by African–American (25.39%), Hispanic (22.3%), and Chinese–American (12.39%). Among White, Chinese–American, and African–American, over 80% of those participants were rare or never consumers, while about half of the Hispanics were medium (23.07%) or heavy (27.55%) consumers. Compared to rare or never and medium consumers, heavy consumers, were similar in age, had less percentage of women, and lower educational and income levels. The average BMI was similar across groups.

**Table 1:** Baseline Characteristics of Multi-Ethnic Study of Atherosclerosis participants by avocado/guacamole consumption (level) (n = 5,794)^a^

	All (n = 5794)	Rare or never (n = 4564)	Medium (n = 683)	Heavy (n = 547)
Avocado (serving per day)	0.04 (0.18)	0.003 (0.01)	0.06 (0.02)	0.31 (0.26)
C-reactive protein (mg/L)	3.69 (5.64)	3.65 (5.52)	3.94 (6.32)	3.76 (5.75)
Interleukin-2 (pg/mL)	996.30 (440.06)	982.77 (438.65)	1011.44 (452.82)	1087.42 (426.16)
Interleukin-6 (pg/mL)	1.54 (1.21)	1.53 (1.22)	1.53 (1.19)	1.67 (1.15)
Total homocysteine (umol/L)	9.33 (3.80)	9.39 (3.94)	8.89 (2.95)	9.39 (3.59)
Fibrinogen antigen (mg/dL)	345.22 (73.67)	345.25 (73.81)	343.04 (72.78)	347.74 (73.72)
Tumor necrosis factor-A soluble receptors (pg/mL)	1380.74 (440.25)	1370.68 (424.23)	1373.65 (451.31)	1412.99 (498.71)
Age (years)	62.25 (10.26)	62.56 (10.20)	60.25 (10.28)	62.14 (10.49)
45–54 (%)	28.29	27.13	35.72	28.70
55–64 (%)	27.58	27.41	28.99	27.24
65–74 (%)	29.65	30.72	23.43	28.52
75–84 (%)	14.48	14.75	11.86	15.54
Female (%)	52.00	51.97	54.17	49.54
Race/ethnicity (%)				
White	39.92	42.75	37.34	19.56
Chinese–American	12.39	14.22	6.44	4.57
African–American	25.39	29.05	12.59	10.79
Hispanic	22.30	13.98	43.63	65.08
Educational level (%)				
Less than high school	17.94	15.03	21.23	38.03
High school	18.16	18.70	14.49	18.28
< 4 years of college	28.53	29.08	28.11	24.50
≥ 4 years of college	35.37	37.20	36.16	19.20
Income level (%)				
< $25,000	31.08	29.54	30.18	45.08
$25,000– < $50,000	28.76	28.70	27.81	30.49
≥ $50,000	40.16	41.76	42.01	24.43
Energy intake (kcals/day)	1673.00 (808.03)	1607.66 (769.44)	1805.61 (858.98)	2033.21 (923.77)
Smoking status (%)				
Never	50.16	50.29	48.46	51.19
Former	37.35	37.38	38.95	35.10
Current	12.49	12.33	12.59	13.71
Alcohol consumption (g/day)	5.17 (12.51)	4.90 (11.95)	7.15 (16.20)	4.94 (11.56)
Physical activity (metabolic equivalent-minutes/week)	1562.56 (2334.58)	1557.98 (2305.35)	1629.62 (2257.84)	1516.95 (2652.36)
Diet quality (Alternative Healthy Eating Index)	42.22 (11.66)	41.49 (11.62)	44.79 (11.66)	44.93 (11.12)
Body mass index (kg/m^2^)	28.28 (5.41)	28.23 (5.44)	28.55 (5.37)	28.41 (5.20)
Underweight/normal (%)	28.46	29.62	23.72	24.68
Overweight (%)	40.08	39.48	41.58	43.14
Obese I/II (%)	27.89	27.32	30.75	29.07
Obese III (%)	3.57	3.57	3.95	3.11
Diabetes type (%)				
Normal	73.14	73.55	74.41	68.07
Impaired fasting glucose	14.22	14.11	14.26	15.05
Untreated diabetes	2.51	2.51	2.35	2.75
Treated diabetes	10.13	9.83	8.97	14.13
Medication(s) (%)—yes ^b^	39.32	40.53	38.65	31.44

^a^Means (standard errors) for continuous variables and percentages for categorical variables. Rare or never = daily serving size ≤ 0.03; Medium = 0.03 < daily serving size < 0.1; Heavy = 0.1 ≤ daily serving size

^b^Medication(s) (hormone replacement therapy, oral steroids, oral anti-inflammatory asthma drugs leukotriene receptor antagonists and inhibitors of lipo-oxygenase, inhaled steroids for asthma, anti-psychotic medications, aspirin) that may influence inflammatory markers

Table 2 presents the results from the three adjusted models of the multivariate analyses. There were no significant differences among the three avocado consumer groups in both models on any inflammatory marker. Results remained similar when waist circumference instead of BMI was added as a covariate.

**Table 2:** Adjusted mean levels (95% confidence interval) of inflammatory markers according to frequency of avocado consumption, Multi-Ethnic Study of Atherosclerosis (Exam 1), United States

Inflammatory marker^b^	Avocado/guacamole consumption^a^			*p* for trend
	Rare or never	Medium	Heavy	
C-reactive protein (mg/L)				
Model 1^c^	1.80 (1.73–1.87)	1.79 (1.64–1.96)	1.65 (1.49–1.84)	0.19
Model 2^d^	2.48 (2.31–2.67)	2.51 (2.26–2.79)	2.47 (2.19–2.77)	0.97
Model 3^e^	2.52 (2.36–2.69)	2.50 (2.26–2.77)	2.54 (2.27–2.83)	0.94
Interleukin-2 (pg/mL)				
Model 1^c^	957.62 (940.12–975.44)	937.40 (897.01–979.61)	950.87 (904.49–999.63)	0.59
Model 2^d^	999.24 (964.29–1035.47)	979.09 (928.16–1032.82)	992.76 (935.49–1052.54)	0.62
Model 3^e^	997.03 (964.35–1030.93)	968.76 (920.92–1019.10)	997.03 (943.16–1053.98)	0.65
Interleukin-6 (pg/mL)				
Model 1^c^	1.24 (1.21–1.27)	1.21 (1.14–1.27)	1.23 (1.16–1.31)	0.68
Model 2^d^	1.57 (1.50–1.63)	1.54 (1.45–1.64)	1.62 (1.51–1.74)	0.52
Model 3^e^	1.57 (1.52–1.64)	1.53 (1.44–1.62)	1.62 (1.52–1.72)	0.73
Total homocysteine (umol/L)				
Model 1^c^	9.03 (8.94–9.12)	8.85 (8.65–9.06)	9.09 (8.85–9.34)	0.90
Model 2^d^	9.19 (9.02–9.37)	9.05 (8.80–9.30)	9.32 (9.03–9.61)	0.74
Model 3^e^	9.18 (9.01–9.35)	9.06 (8.81–9.31)	9.33 (9.05–9.63)	0.52
Fibrinogen (mg/dL)				
Model 1^c^	340.16 (337.71–342.63)	336.87 (331.39–342.44)	334.31 (328.01–340.74)	0.06
Model 2^d^	357.99 (353.28–362.76)	355.83 (348.92–362.87)	354.23 (346.54–362.09)	0.25
Model 3^e^	358.62 (354.16–363.14)	355.19 (348.56–361.95)	353.91 (346.58–361.40)	0.11
Tumor necrosis factor-A soluble receptors (pg/mL)				
Model 1^c^	1366.21 (1348.62–1384.03)	1347.97 (1306.79–1390.45)	1324.76 (1278.97–1372.19)	0.09
Model 2^d^	1465.82 (1430.73–1501.77)	1440.80 (1389.07–1494.45)	1426.53 (1369.70–1485.71)	0.10
Model 3^e^	1468.95 (1436.59–1502.04)	1457.09 (1408.27–1507.60)	1452.16 (1399.20–1507.13)	0.45

^a^Rare or never = daily serving size ≤ 0.03; Medium = 0.03 < daily serving size < 0.1; Heavy = 0.1 ≤ daily serving size

^b^Antilogs of adjusted mean levels of log-transformed inflammatory markers

^c^Model 1 adjusted for age, sex, race/ethnicity, field center, educational level, income, energy intake (kcals/day). Sample sizes for each inflammatory markers varied because of missing covariates: C-reactive protein (n = 5336); Interleukin-2 (n = 2304); Interleukin-6 (n = 5240); Total homocysteine (n = 5354); Fibrinogen (n = 5337); Tumor Necrosis Factor-A Soluble Receptors (n = 2309)

^d^Model 2 further adjusted for smoking status, alcohol consumption, physical activity, diet quality (AHEI), BMI, Exam 1 Diabetes Type, medication(s) that may influence inflammatory markers. Sample sizes for each inflammatory markers varied because of missing covariates: C-reactive protein (n = 5115); Interleukin-2 (n = 2220); Interleukin-6 (n = 5025); Total homocysteine (n = 5132); Fibrinogen (n = 5116); Tumor Necrosis Factor-A Soluble Receptors (n = 2226)

^e^Model 3 adjusted for covariates selected from the stepwise selection procedure (significance level of 0.05 was used as a criterion for variables to enter or stay in the model). C-reactive protein—adjusted for age, sex, race/ethnicity, smoking status, AHEI, BMI, Exam 1 Diabetes Type. Interleukin-2—adjusted for age, sex, race/ethnicity, income, smoking status, alcohol consumption, BMI, Exam 1 Diabetes Type. Interleukin-6—adjusted for age, sex, race/ethnicity, income, smoking status, physical activity, BMI, Exam 1 Diabetes Type. Total homocysteine: age, sex, race/ethnicity, field center, income, smoking status, alcohol consumption, AHEI, BMI, Exam 1 Diabetes Type. Fibrinogen—adjusted for age, sex, race/ethnicity, field center, income, smoking status, alcohol consumption, physical activity, BMI, Exam 1 Diabetes Type. Tumor Necrosis Factor-A Soluble Receptors—adjusted for age, sex, race/ethnicity, smoking status, alcohol consumption, BMI, Exam 1 Diabetes Type

## Discussion

In this cross-sectional study, we did not find that consumption of avocado/guacamole was associated with levels of inflammatory markers in our primary analysis.

Our analysis showed no significant difference in inflammatory markers measured among the three consumer groups. This is comparable to the findings observed in previous studies [21–27]. Some shorter-term studies found a potential benefit of avocado on some inflammatory markers. For instance, in one clinical trial [27], 51 healthy men and women with overweight and obesity were assigned to either a hypocaloric diet (control group) or a hypocaloric diet with one daily Hass avocado (intervention group) over 12 weeks. While the researchers did not observe significant changes in IL-6 and other markers of inflammation, they noted a trend of decreased IL-1β and CRP with a hypocaloric diet plus avocado group (vs. hypocaloric diet), and the results reached significance in an acute study [25]. Li et al. found that when consuming a burger patty with avocado, compared to a burger alone, decreased activation of the NF-kappa B inflammatory pathway at 3 h post-meal [25]. IL-6 was significantly higher at 4 h post-meal in the burger group but not in the burger topped with the avocado group [25]. The mixed results may be due to the smaller sample sizes (e.g., not powered to examine this secondary outcome), different populations used in each study, or different assessed inflammatory markers. It is also possible that avocado may have a more prominent short (vs. longer-term) effect on inflammatory markers. Moreover, there may be different effects on acute vs. chronic inflammation, but no set definition or inflammatory markers exist to differentiate them [37]. Also, ranges of inflammatory markers can vary widely depending on several factors, such as genetic, even among healthy adults [5].

In our study, the FFQ used in the MESA cohort combined avocado and guacamole as one question. Thus, that may dilute the actual impact of avocado since guacamole contains a combination of ingredients besides avocado. Also, the average daily servings for medium and heavy consumers were 0.06 and 0.31, respectively, which were low compared to the aforementioned clinical trials. Perhaps these low levels of avocado consumption did not meet the threshold to produce any significant changes in outcomes. It is also possible that looking at dietary patterns (vs. single food) may have greater effects. For example, Nettleton et al. [36] examined the association between dietary patterns and markers of inflammation in the Multi-Ethnic Study of Atherosclerosis (MESA). They reported that the whole grains and fruit pattern was inversely related to markers of inflammation, such as homocysteine, IL-6, and CRP (*P* for trend ≤ 0.001) [36]. Mediterranean diet also showed similar effects [3]. A systematic review and meta-analysis of 17 intervention trials found that the Mediterranean diet was associated with significant decreases in CRP (weight mean differences: − 0.98 mg/l, 95% CI − 1.48 to − 0.49) and IL-6 (weight mean differences: − 0.42 pg/ml, 95% CI − 0.73 to − 0.11) [38]. The nutrient profile of an avocado aligns well with those dietary patterns. Although examining dietary patterns is superior in considering the synergistic effects of different food components, using a single food or nutrient approach can provide practical insights for practitioners. For example, patients are often interested to know whether to increase or decrease the consumption of a particular food item within a certain dietary pattern to improve their health.

Our observational study confirms results from smaller studies and clinical trials that although avocados are high in fat, consumption is not associated with increased levels of inflammatory markers. High-fat diets, in general, have been linked to inflammation and a host of other diseases, but not all fatty acids have similar health effects [12–18]. For instance, fatty acids play a crucial role in inflammatory activities through toll-like receptor 4 (TLR4), a transmembrane protein that recognizes pathogens and stimulates inflammatory responses [16]. Lee et al. found that saturated fatty acids triggered inflammation through TLR4, but monounsaturated and polyunsaturated fatty acids did not activate the signaling of TLR4 [17]. One serving size of an avocado (i.e., 1/3 medium avocado or 50 g) contains 8 g of total fat, 1 g of polyunsaturated fatty acids, and 5 g of monounsaturated fatty acids [11]. This may explain why we did not find an increase in inflammatory markers among the three consumer groups: ~ 75% of the fatty acids in avocados are unsaturated [11]. This is consistent with other studies that used foods with similar compositions (e.g., olives and olive oil, which are rich in oleic acid). For example, Jiménez-Gómez et al. found that a meal with butter, but not walnut or olive oil, induced higher mRNA expression of soluble tumor necrosis factor-alpha [20]. Perez-Martinez et al. also reported comparable findings in their randomized crossover trial. They found that compared to a Western diet, a Mediterranean diet with olive oil did not increase nuclear transcription factor kappaB, which serves an essential role in the inflammatory process [40]. These studies further confirm the different effects that each type of fatty acid has on inflammation.

There are several strengths of this study. First, we used the MESA cohort, which included a large and diverse population across six different sites. Second, we adjusted for several confounders in our final model to better understand the association between avocado/guacamole consumption and inflammatory markers. Third, avocado/guacamole consumption was assessed with an FFQ, which represents usual intake and captures sporadically consumed foods like the avocado [41]. However, several limitations should be noted when interpreting the results of this study. First, we used a cross-sectional observational study design, which cannot examine causality and may be prone to potential residual confounding effects. Second, MESA Exam 1 took place between 2000 and 2002, so it may not accurately represent current avocado consumption, which has increased from an annual 5 pounds to 9 pounds *per capita* from 2011 to 2020. Collecting multiple FFQs at different time points may provide more accurate consumption data. Third, there may be some self-reported errors with the FFQ, resulting in misclassification. Also, the FFQ combined avocado and guacamole as one question. Thus, we were not able to parse out individual effects. The proportion of different ingredients, such as avocado and salt, in guacamole may vary, but the FFQ did not capture this data. Lastly, MESA enrolled women and men (45–84 years old) without clinical cardiovascular disease at baseline across six study sites in the United States, so this cohort may not represent the general population [28].

In conclusion, there were no significant differences in any inflammatory markers measured among the three avocado/guacamole consumer groups. This supports the idea that not all foods high in fat are associated with inflammation. In this example, avocados contain 8 g of total fat per serving (monounsaturated or polyunsaturated fatty acids constitute 75% of the total fat) and we found no effects on inflammation among MESA participants. Thus, evaluating the fatty acid composition is crucial rather than looking solely at the total fat content. However, this cohort may not be representative of the US population and avocado/guacamole consumers in this cohort were 71% minority. Therefore, future studies are warranted to investigate this association in other populations and with a recent dietary assessment of avocado/guacamole to represent current intake.

## Data Availability

Available.

## Code availability (software application or custom code)

Available.

## Declarations

### Additional declarations for articles in life science journals that report the results of studies involving humans and/or animals

N/A.

## References

[1] Childs CE, Calder PC, Miles EA (2019). Diet and immune function. Nutrients.

[2] Iddir M, Brito A, Dingeo G, Fernandez Del Campo SS, Samouda H, La Frano MR, Bohn T (2020). Strengthening the immune system and reducing inflammation and oxidative stress through diet and nutrition: considerations during the COVID-19 crisis. Nutrients.

[3] Casas R, Sacanella E, Estruch R (2014). The immune protective effect of the Mediterranean diet against chronic low-grade inflammatory diseases. Endocr Metab Immune Disord Drug Targets.

[4] Luo Y, Liu M (2016). Adiponectin: a versatile player of innate immunity. J Mol Cell Biol.

[5] Calder PC, Ahluwalia N, Albers R, Bosco N, Bourdet-Sicard R, Haller D, Holgate ST, Jonsson LS, Latulippe ME, Marcos A, Moreines J, M'Rini C, Muller M, Pawelec G, van Neerven RJ, Watzl B, Zhao J (2013). A consideration of biomarkers to be used for evaluation of inflammation in human nutritional studies. Br J Nutr.

[6] Gombart AF, Pierre A, Maggini S (2020). A review of micronutrients and the immune system-working in harmony to reduce the risk of infection. Nutrients.

[7] Dreher ML (2018). Whole fruits and fruit fiber emerging health effects. Nutrients.

[8] Calder PC, Carr AC, Gombart AF, Eggersdorfer M (2020). Optimal nutritional status for a well-functioning immune system is an important factor to protect against viral infections. Nutrients.

[9] Venter C, Eyerich S, Sarin T, Klatt KC (2020). Nutrition and the immune system: a complicated tango. Nutrients.

[10] Hosseini B, Berthon BS, Saedisomeolia A, Starkey MR, Collison A, Wark PAB, Wood LG (2018). Effects of fruit and vegetable consumption on inflammatory biomarkers and immune cell populations: a systematic literature review and meta-analysis. Am J Clin Nutr.

[11] Dreher ML, Davenport AJ (2013). Hass avocado composition and potential health effects. Crit Rev Food Sci Nutr.

[12] Duan Y, Zeng L, Zheng C, Song B, Li F, Kong X, Xu K (2018). Inflammatory links between high fat diets and diseases. Front Immunol.

[13] Payette C, Blackburn P, Lamarche B, Tremblay A, Bergeron J, Lemieux I, Despres JP, Couillard C (2009). Sex differences in postprandial plasma tumor necrosis factor-alpha, interleukin-6, and C-reactive protein concentrations. Metabolism.

[14] Nappo F, Esposito K, Cioffi M, Giugliano G, Molinari AM, Paolisso G, Marfella R, Giugliano D (2002). Postprandial endothelial activation in healthy subjects and in type 2 diabetic patients: role of fat and carbohydrate meals. J Am Coll Cardiol.

[15] Lundman P, Boquist S, Samnegard A, Bennermo M, Held C, Ericsson CG, Silveira A, Hamsten A, Tornvall P (2007). A high-fat meal is accompanied by increased plasma interleukin-6 concentrations. Nutr Metab Cardiovasc Dis.

[16] Fritsche KL (2015). The science of fatty acids and inflammation. Adv Nutr.

[17] Lee JY, Sohn KH, Rhee SH, Hwang D (2001). Saturated fatty acids, but not unsaturated fatty acids, induce the expression of cyclooxygenase-2 mediated through Toll-like receptor 4. J Biol Chem.

[18] Teng KT, Chang CY, Chang LF, Nesaretnam K (2014). Modulation of obesity-induced inflammation by dietary fats: mechanisms and clinical evidence. Nutr J.

[19] Masson CJ, Mensink RP (2011). Exchanging saturated fatty acids for (n-6) polyunsaturated fatty acids in a mixed meal may decrease postprandial lipemia and markers of inflammation and endothelial activity in overweight men. J Nutr.

[20] Jimenez-Gomez Y, Lopez-Miranda J, Blanco-Colio LM, Marin C, Perez-Martinez P, Ruano J, Paniagua JA, Rodriguez F, Egido J, Perez-Jimenez F (2009). Olive oil and walnut breakfasts reduce the postprandial inflammatory response in mononuclear cells compared with a butter breakfast in healthy men. Atherosclerosis.

[21] Khor A, Grant R, Tung C, Guest J, Pope B, Morris M, Bilgin A (2014). Postprandial oxidative stress is increased after a phytonutrient-poor food but not after a kilojoule-matched phytonutrient-rich food. Nutr Res.

[22] Wang L, Bordi PL, Fleming JA, Hill AM, Kris-Etherton PM (2015). Effect of a moderate fat diet with and without avocados on lipoprotein particle number, size and subclasses in overweight and obese adults: a randomized, controlled trial. J Am Heart Assoc.

[23] Pieterse Z, Jerling JC, Oosthuizen W, Kruger HS, Hanekom SM, Smuts CM, Schutte AE (2005). Substitution of high monounsaturated fatty acid avocado for mixed dietary fats during an energy-restricted diet: effects on weight loss, serum lipids, fibrinogen, and vascular function. Nutrition.

[24] Scott TM, Rasmussen HM, Chen O, Johnson EJ (2017). Avocado consumption increases macular pigment density in older adults: a randomized controlled trial. Nutrients.

[25] Li Z, Wong A, Henning SM, Zhang Y, Jones A, Zerlin A, Thames G, Bowerman S, Tseng CH, Heber D (2013). Hass avocado modulates postprandial vascular reactivity and postprandial inflammatory responses to a hamburger meal in healthy volunteers. Food Funct.

[26] Park E, Edirisinghe I, Burton-Freeman B (2018). Avocado fruit on postprandial markers of cardio-metabolic risk: a randomized controlled dose response trial in overweight and obese men and women. Nutrients.

[27] Henning SM, Yang J, Woo SL, Lee RP, Huang J, Rasmusen A, Carpenter CL, Thames G, Gilbuena I, Tseng CH, Heber D, Li Z (2019). Hass avocado inclusion in a weight-loss diet supported weight loss and altered gut microbiota: a 12-week randomized parallel-controlled trial. Curr Dev Nutr.

[28] Bild DE, Bluemke DA, Burke GL, Detrano R, Diez Roux AV, Folsom AR, Greenland P, Jacob DR, Kronmal R, Liu K, Nelson JC, O'Leary D, Saad MF, Shea S, Szklo M, Tracy RP (2002). Multi-Ethnic Study of Atherosclerosis: objectives and design. Am J Epidemiol.

[29] Jiang R, Jacobs DR, Mayer-Davis E, Szklo M, Herrington D, Jenny NS, Kronmal R, Barr RG (2006). Nut and seed consumption and inflammatory markers in the multi-ethnic study of atherosclerosis. Am J Epidemiol.

[30] Hass Avocado Board (2019) Avocados Tracking 2019|General Sample Segment Report

[31] Cushman M, Cornell ES, Howard PR, Bovill EG, Tracy RP (1995). Laboratory methods and quality assurance in the cardiovascular health study. Clin Chem.

[32] Dong X, Li S, Chen J, Li Y, Wu Y, Zhang D (2020). Association of dietary omega-3 and omega-6 fatty acids intake with cognitive performance in older adults: National Health and nutrition examination Survey (NHANES) 2011–2014. Nutr J.

[33] McCullough ML, Feskanich D, Stampfer MJ, Giovannucci EL, Rimm EB, Hu FB, Spiegelman D, Hunter DJ, Colditz GA, Willett WC (2002). Diet quality and major chronic disease risk in men and women: moving toward improved dietary guidance. Am J Clin Nutr.

[34] Moore LV, Diez Roux AV, Nettleton JA, Jacobs DR (2008). Associations of the local food environment with diet quality–a comparison of assessments based on surveys and geographic information systems: the multi-ethnic study of atherosclerosis. Am J Epidemiol.

[35] Aiello AE, Diez-Roux A, Noone AM, Ranjit N, Cushman M, Tsai MY, Szklo M (2009). Socioeconomic and psychosocial gradients in cardiovascular pathogen burden and immune response: the multi-ethnic study of atherosclerosis. Brain Behav Immun.

[36] Nettleton JA, Steffen LM, Mayer-Davis EJ, Jenny NS, Jiang R, Herrington DM, Jacobs DR (2006). Dietary patterns are associated with biochemical markers of inflammation and endothelial activation in the Multi-Ethnic Study of Atherosclerosis (MESA). Am J Clin Nutr.

[37] Calder PC, Ahluwalia N, Brouns F, Buetler T, Clement K, Cunningham K, Esposito K, Jonsson LS, Kolb H, Lansink M, Marcos A, Margioris A, Matusheski N, Nordmann H, O'Brien J, Pugliese G, Rizkalla S, Schalkwijk C, Tuomilehto J, Warnberg J, Watzl B, Winklhofer-Roob BM (2011). Dietary factors and low-grade inflammation in relation to overweight and obesity. Br J Nutr.

[38] Schwingshackl L, Hoffmann G (2014). Mediterranean dietary pattern, inflammation and endothelial function: a systematic review and meta-analysis of intervention trials. Nutr Metab Cardiovasc Dis.

[39] Christ A, Lauterbach M, Latz E (2019). Western diet and the immune system: an inflammatory connection. Immunity.

[40] Perez-Martinez P, Lopez-Miranda J, Blanco-Colio L, Bellido C, Jimenez Y, Moreno JA, Delgado-Lista J, Egido J, Perez-Jimenez F (2007). The chronic intake of a Mediterranean diet enriched in virgin olive oil, decreases nuclear transcription factor kappaB activation in peripheral blood mononuclear cells from healthy men. Atherosclerosis.

[41] Shim JS, Oh K, Kim HC (2014). Dietary assessment methods in epidemiologic studies. Epidemiol Health.

